# TOGging up microtubule ends: The plant-specific microtubule minus-end tracking SPR2 has a unique TOG domain

**DOI:** 10.1093/plcell/koad323

**Published:** 2023-12-22

**Authors:** Renuka Kolli

**Affiliations:** Assistant Features Editor, The Plant Cell, American Society of Plant Biologists; Sainsbury Laboratory, University of Cambridge, Cambridge, UK

Cortical microtubule arrays associated with the plasma membrane are crucial for cell shape and intracellular trafficking. They are distinctly arranged in different cell types and dynamically rearranged in response to various internal and external stimuli. Individual microtubules within the arrays exhibit hybrid treadmilling with the minus-ends predominantly pausing or depolymerizing and the plus-ends alternating between polymerizing and depolymerizing phases. Microtubule dynamics are regulated by a variety of microtubule-binding proteins, including stabilizers, destabilizers, capping proteins, and bundlers/cross-linkers. Regulation of microtubule dynamics is especially important at the many free minus-ends of the microtubule arrays and at the new minus-ends generated rapidly by severing during the creation of new microtubule arrays. In animals, proteins belonging to the CAMSAP/Patronin/Nezha family regulate microtubule minus-end dynamics. Plants lack CAMSAP/Patronin/Nezha proteins but instead have SPIRAL2 (SPR2). SPR2 tracks and stabilizes the microtubule minus-ends by reducing the depolymerization rate, thereby facilitating light-induced reorientation of the microtubule arrays (Fan et al. 2018; Leong et al. 2018; Nakamura et al. 2018). However, the structural features of SPR2 that determine its regulatory role at the microtubule minus-end remained unknown.

The Arabidopsis SPR2 N-terminus was predicted to have 5 Huntington, Elongation factor 3, phosphatase 2A, Target of rapamycin 1 (HEAT) repeats followed by a basic region. In this issue, **Yuanwei Fan and coauthors** (Fan et al. 2023) present the X-ray crystallography-based structure of the SPR2 N-terminal domain at 2.5 Å resolution displaying 7 HEAT repeats that constitute a unique Tumor Overexpressed Gene (TOG) domain (see Fig. 1A). TOG domains consist of 6 HEAT repeats that have been well-characterized in the XMAP215 and CLASP family proteins, which mainly target and regulate microtubule plus-ends in fungi, animals, and plants. The structure of yeast (*Saccharomyces cerevisiae*) XMAP215 protein, called Stu2, bound to αβ-tubulin was previously determined (Ayaz et al. 2014; see Fig. 1B). Structural alignment of the SPR2 TOG domain to the Stu2 TOG2 domain and sequence alignment of SPR2 homologs revealed that several SPR2 residues, at positions equivalent to tubulin-binding Stu2 residues, are conserved across plant species. Therefore, SPR2's extended TOG architecture might bind tubulin in a conformation different from that bound by Stu2 TOG2. Initial lattice binding of SPR2 likely promotes its ultimate localization at the microtubule minus-end. Results from in vitro microtubule dynamics reconstitution experiments and live imaging of hypocotyl epidermal cells indicate that the basic region following the TOG domain is important for binding the negatively charged microtubule lattice.

**Figure 1.**
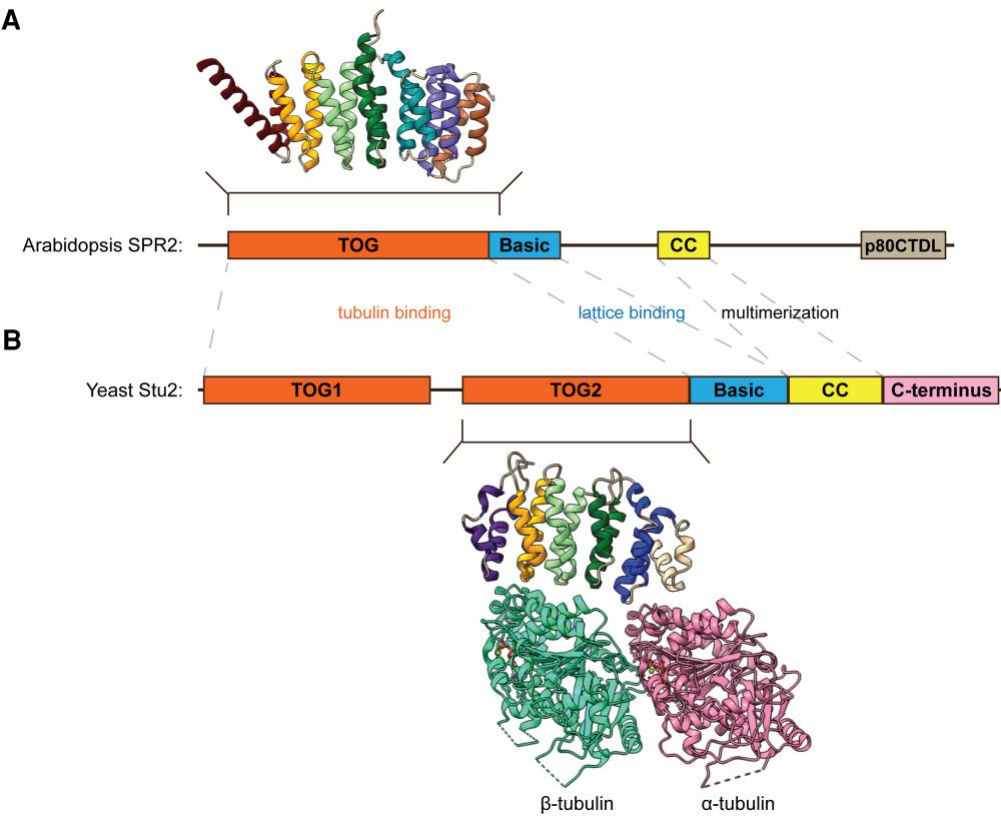
Microtubule-binding proteins with similar structures but targeting the opposite ends. **A)** The minus-end targeting SPR2's TOG domain structure. **B)** The plus-end targeting Stu2 with the crystal structure of TOG2 in complex with tubulin dimer. CC = coiled-coil; p80CTDL = Katanin p80 C-terminal domain-like. Figure credit: R. Kolli. Protein structures obtained from the PDB database (https://www.rcsb.org/) were adapted using ChimeraX (Meng et al. 2023).

Using size exclusion chromatography with multi-angle light scattering analysis, the authors show that SPR2 forms a tetramer via a coiled-coil domain in the middle of the protein. SPR2 tetramerization enhances the microtubule binding strength. Moreover, it allows simultaneous binding of the microtubule lattice and soluble tubulin as demonstrated by a total internal reflection fluorescence microscopy-based assay. The authors show that a truncated SPR2 containing the TOG domain, the basic region, and the coiled-coil domain can target and stabilize microtubule minus-ends similar to the full-length SPR2 both in vitro and in vivo. Furthermore, expression of the truncated SPR2 in the Arabidopsis *spr2-2* mutant rescued its right-handed helical growth phenotype. Hence the SPR2 C-terminal domain is dispensable for microtubule minus-end stabilization. However, it might be important for regulating microtubule severing as the crystal structure resembles the Katanin p80 C-terminal domain (Ohno et al. 2023).

In animals, a C-terminal CKK (CAMSAP1, KIAA1078, and KIAA1543) domain in the CAMSAP/Patronin/Nezha family proteins allows microtubule minus-end targeting. However, plants appear to have co-opted the TOG domain, generally found in microtubule plus-end regulators, into SPR2 to regulate microtubule minus-end dynamics (see Fig.). How SPR2 reduces microtubule minus-end depolymerization rate at the molecular level remains an open question. Future research could investigate whether tetrameric SPR2 provides ready access to soluble tubulin at the minus-end for its stabilization. Also, it would be interesting to know whether the unique TOG domain of SPR2 binds and stabilizes a tubulin conformation that is specific to the microtubule minus-end.

## Dive Curated Terms

The following phenotypic, genotypic, and functional terms are of significance to the work described in this paper:

SPR2 Gramene: AT4G27060
SPR2 Araport: AT4G27060

protein CHEBI: CHEBI:36080

## References

[koad323-B1] Ayaz P , Munyoki S, Geyer EA, Piedra F-A, Vu ES, Bromberg R, Otwinowski Z, Grishin NV, Brautigam CA, Rice LM. A tethered delivery mechanism explains the catalytic action of a microtubule polymerase. Elife. 2014:3:e03069. 10.7554/eLife.03069 25097237 PMC4145800

[koad323-B2] Fan Y , Bilkey N, Bolhuis DL, Slep KC, Dixit R. A divergent tumor overexpressed gene domain and oligomerization contribute to SPIRAL2 function in stabilizing microtubule minus ends. Plant Cell. 2024:36 (4):1056–1071. 10.1093/plcell/koad294 38011314 PMC10980349

[koad323-B3] Fan Y , Burkart GM, Dixit R. The Arabidopsis SPIRAL2 protein targets and stabilizes microtubule minus ends. Curr Biol. 2018:28(6):987–994e983. 10.1016/j.cub.2018.02.014 29526586 PMC5860991

[koad323-B4] Leong SY , Yamada M, Yanagisawa N, Goshima G. SPIRAL2 stabilises endoplasmic microtubule minus ends in the moss physcomitrella patens. Cell Struct Funct. 2018:43(1):53–60. 10.1247/csf.18001 29445053

[koad323-B5] Meng EC , Goddard TD, Pettersen EF, Couch GS, Pearson ZJ, Morris JH, Ferrin TE. UCSF chimerax: tools for structure building and analysis. Protein Sci. 2023:32(11):e4792. 10.1002/pro.4792 37774136 PMC10588335

[koad323-B6] Nakamura M , Lindeboom JJ, Saltini M, Mulder BM, Ehrhardt DW. SPR2 protects minus ends to promote severing and reorientation of plant cortical microtubule arrays. J Cell Biol. 2018:217(3):915–927. 10.1083/jcb.201708130 29339437 PMC5839793

[koad323-B7] Ohno M , Higuchi Y, Hayashi I. Crystal structure of the C-terminal domain of the plant-specific microtubule-associated protein Spiral2. Acta Crystallogr F Struct Biol Commun. 2023:79(1):17–22. 10.1107/S2053230X22011815 36598352 PMC9813970

